# HS-SPME-GC/MS Analysis for Revealing Carob’s Ripening

**DOI:** 10.3390/metabo12070656

**Published:** 2022-07-15

**Authors:** Panagiota Fella, Kyriaki Kaikiti, Marinos Stylianou, Agapios Agapiou

**Affiliations:** Department of Chemistry, University of Cyprus, Nicosia 1678, Cyprus; fella.panagiota@ucy.ac.cy (P.F.); kaikiti.kyriaki@ucy.ac.cy (K.K.); stylianou.a.marinos@ucy.ac.cy (M.S.)

**Keywords:** carob fruit, VOCs, ripening process, odor, smell

## Abstract

Carob’s recognized nutritional and medicinal value next to its unique agriculture importance is associated with an array of social, economic, and cultural activities. The carob fruit is popular for its intense aroma due to the emitted volatile organic compounds (VOCs). The composition of VOCs released from carob fruits changes during ripening, rendering it a non-invasive tool for the determination of the ripening period and freshness of the fruit. Therefore, headspace solid-phase microextraction gas chromatography/mass spectrometry (HS-SPME-GC/MS) was applied to reveal the respective gaseous signal molecules related to fruit maturity. The sampling was implemented during weeks 26–36 from five different locations in Cyprus. Additionally, the gaseous emissions of total VOCs (TVOCs) and carbon dioxide (CO_2_) were recorded next to the moisture content of the fruit. The major chemical classes in the ripening are acids, followed by esters, and ketones. More specifically, the most abundant VOCs during ripening are propanoic acid, 2-methyl-(isobutyric acid), 2-heptanone, propanoic acid, 2-methyl-, 2-methylbutyl ester, acetic acid, methyl isobutyrate, propanoic acid, 2-methyl-, 3-methylbutyl ester, 2-pentanone, butanoic acid and propanoic acid, 2-methyl-ethyl ester. Finally, CO_2_ emissions and moisture content showed a rapid decline until the 31st week and then stabilized for all examined areas. The methodology revealed variations in VOCs’ profile during the ripening process.

## 1. Introduction

The carob tree (*Ceratonia siliqua* L.) belongs to the family of Leguminosae, in the genus of Ceratonia. The thermophilus nature of the tree allows it to thrive in environments with mild and drought climate conditions, such as in most Mediterranean countries [1]. Carob cultivation in Cyprus dates back to the first centuries and is inextricably linked with the culture and society of the island. As one of the most important exportable products, it also plays an important role in the agricultural economy of the country [2].

Biogenic volatile organic compounds (BVOCs) are major chemical components of the natural environment, as they contribute to the communication between plants, insects, hosts, soil organisms, etc. The part of the earth system that includes all ecosystems and living organisms in the atmosphere and on land, acts as the head source of BVOCs. Furthermore, BVOCs are released in the rhizosphere at low concentrations to regulate plants’ growth, well-being, resistance, and nutrient uptake [3,4]. In general, flowers and fruits release a wide variety of BVOCs, with the emission rates peaking during ripening. On the other hand, BVOCs contribute to troposphere chemistry triggering (directly or indirectly) the production of air pollutants and greenhouse gases, as well as increasing acidity and aerosol production [5,6,7].

Fruit color and other organoleptic characteristics are evidence of fruit maturity. The ripening of the carob fruit takes almost a year and maturity is judged by the color of the carob pod (from green bean to dark brown with hard texture pulp) and the respective dehydration. According to Kyriacou et al. [8], the fruit respiration rate declines during the ripening process of the carobs; the same applies also to the phenolic content, tannins, hydrolysable tannins, catechins, and flavonol glycosides. Depending on the carob variety and local climate, the fruits ripen from early August to late October. During British colonization, carob harvesting in Cyprus was governed by a law which stipulated the beginning date (usually the end of August) for the carob harvest [9]; this was a protective step for the mills to avoid the acceptance of un-matured fruits and the extra charge due to their potential moisture. Collection of carobs without permission and beyond the dedicated period was illegal and punished by the issuing of a fine or even imprisonment.

The quality and shelf life of fruits are significantly affected by the ripening stage at the time of harvesting. Monitoring the emitted VOCs during fruit ripening is important for fruit management as VOCs play an important role in the taste and aroma, which influence consumer choices [10]. The amount and types of VOCs change due to certain factors such as harvest time and post-harvest storage conditions [11,12].

Several analytical methods were reported for detecting VOCs in fruits and vegetables. These can be classified into on-line (near real-time) and off-line (non real-time) methods. Despite their advantages (immediate results), the on-line methods such as the e-noses [13], and proton-transfer-reaction time-of-flight/mass spectrometer (PTR-TOF/MS) [14,15] lack spectral libraries. On the other hand, the off-line methods (e.g., solid-phase microextraction gas chromatography/mass spectrometry (SPME-GC/MS) [16], thermal desorption-GC/MS (TD-GC/MS), etc.) facilitates the pre-concentration of the sample before the analysis. SPME is a fast and powerful solvent-free analytical technique [17]; in combination with the GC/MS, it constitutes a reliable, efficient, sensitive, and low-cost analytical effective solution. Until now, the HS-SPME-GC/MS method was used for the decoding of the aroma profile emitted by various parts of the carob tree (e.g., powder, flowers, fruits, etc.), followed by the results of chemometric processing [18,19,20]. In addition, it was applied with a flame ionization detector (HS-SPME-GC/MS and FID) to monitor the VOCs’ changes during the ripening of ‘*Nanguoli*’ pears, where 43 VOCs were associated with ripening [21]. Additionally, Liu et al. [22] used GC with ion mobility spectrometry (GC-IMS) to determine the changes in VOCs’ profile during the ripening of avocado, where 30 VOCs were detected. Furthermore, PTR-TOF/MS was applied for VOC analyses of four tropical fruits (avocado, banana, mango, and mangosteen) during maturation [11]. Additionally, F. Nouri et al. [23] determined 17 VOCs during the ripening stages of mango using an e-nose. Moreover, D. Obenland et al. [24] investigated the respective changes during the *Hass* avocado ripening through the SPME-GC/MS analysis and showed that the abundances of hexanal, (E)-2-hexenal, and 2,4-hexadienal declined during maturation, whereas that of acetaldehyde, methyl acetate, pentanal, and β-myrcene increased over time. Thus, Table 1 summarises several studies focusing on the changes of VOCs during fruit ripening.

Nevertheless, limited knowledge is available in the literature on monitoring carobs’ aroma synthesis towards ripening. After harvest, carob fruits are delivered to carob mills for future processing. Independently of the maturity status, moisture content, and variety of the fruit, carobs are accumulated and stored for several months in big warehouses. Therefore, the present study aimed to monitor carob maturation in five different villages of Cyprus using the HS-SPME-GC/MS method through their VOCs signals. Additionally, the emission rates of carbon dioxide (CO_2_) and humidity of the carob fruit were analyzed.

## 2. Results and Discussion

Fruit coloring was previously employed to describe carobs’ different ripening stages. In particular, Kyriacou et al. [8], divided the maturity of carobs into six critical stages (RSI-RS6: fully developed green, dull green, breaker, green pedicel, ripe fully dark, and late ripe fruit), whereas Ben Othmen et al. [31] reported only three stages of carob pods’ maturation (unripe, mid-ripe, and ripe stage).

**Figure 1:**
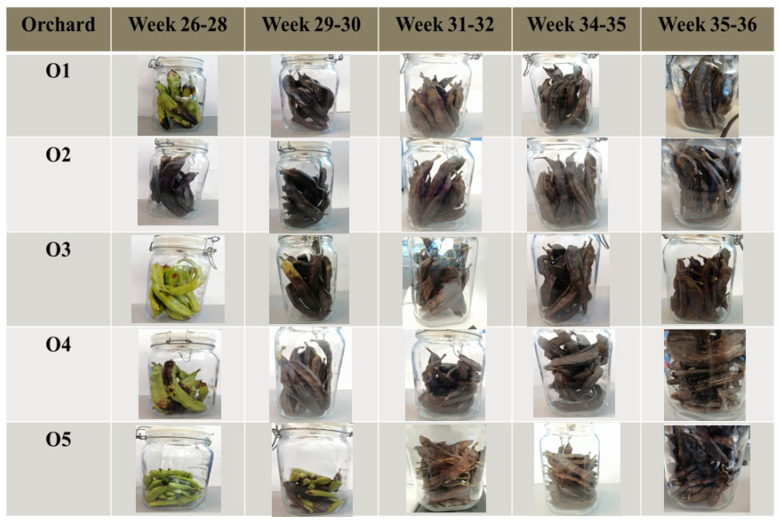
Stages of carob maturation of 5 orchards, from the unripe (green color) to the ripe (dark brown color) stage.

Figure 1 shows the carob fruit samples each week from the five different orchards. The dark brown color of the carob pods indicates the ripening of the fruit. For almost all orchards (O1, O3, O4, O5), between the 26th and 28th week of the examined period, the carobs were almost immature due to the green color except in the case of the orchard O2. From the 29th week of the examined period, the examined samples began ripening, reaching a dark brown color in the last week of the examination period.

### 2.1. Effect of Maturation on the VOCs Profile

HS-SPME-GC/MS analysis highlighted carobs’ main ripening chemical classes from five different orchards (W35-W36). According to Krokou et al., the respective VOCs were emitted in the low ppb_v_ values [19]. The most abundant acids were propanoic acid, 2-methyl- (isobutyric acid), acetic acid, and butanoic acid. The importance of the three latter acids in carob’s aroma profile was highlighted earlier, despite the different geographical origins and fruit cultivation (Cyprus, Spain, and Italy) [19]. In this respect, the dominant presence of isobutyric acid in both carob fruit and powder was noticed, indicating the negative effect of the roasting process on its concentration [18]. The most abundant alcohols and furans were ethanol and furan 2-methyl-, respectively. Note that the presence of ethanol was especially reported in the carob flower [18]. Additionally, the major aldehydes/ketones were butanal 3-methyl-, 2-heptanone, and 2-pentanone. Furthermore, the most abundant esters were propanoic acid, 2-methyl-, 2-methylbutyl ester (isobutyric acid, 2-methylbutyl ester), methyl isobutyrate, propanoic acid, 2-methyl-, 3-methylbutyl ester, propanoic acid, 2-methyl-, ethyl ester and hexanoic acid methyl ester. The wide presence of esters is indicative of the fruit’s rich aroma profile, as they are the second strongest chemical class after acids. Finally, the most abundant hydrocarbons were octane and limonene (cyclic monoterpene). Regardless of the chemical group, the most important VOCs, according to their average values during the examined maturation period (Table 2), were propanoic acid, 2-methyl- (40.54), 2-heptanone (13.74), propanoic acid, 2-methyl-, 2-methylbutyl ester (13.19), acetic acid (6.06), methyl isobutyrate (4.80), propanoic acid, 2-methyl-, 3-methylbutyl ester (4.29), 2-pentanone (4.06), butanoic acid (3.97), propanoic acid, 2-methyl-, ethyl ester (3.37), and hexanoic acid methyl ester (3.21).

**Figure 2:**
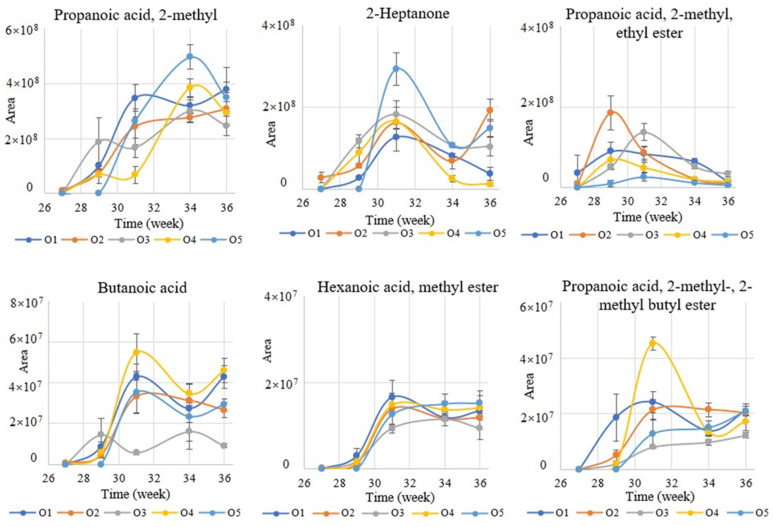
The effect of carob maturation of propanoic acid, 2-methyl- (sour/cheesy odor), 2-heptanone (fruity/spicy odor), propanoic acid, 2-methyl-, ethyl ester- (fruity/sweet odor), butanoic acid (cheesy/buttery odor), hexanoic acid methyl ester (fruity/pineapple odor), and propanoic acid, 2-methyl-, 2-methylbutyl ester for each week of the examined period. ψ-axis (area) corresponds to AUC.

As the main compounds (greater abundance), some of the respective VOCs were selected for further study of their ripeness profile in each week of the analysis. Figure 2 presents the selected VOC trends per week for different villages.

Overall, in the cases of the six selected VOCs, there were some fluctuations in the examined periods, with the highest abundance occurring around the 31st week. More specifically, propanoic acid, 2-methyl-(isobutyric acid) increased significantly in the 31st and 34th week of the examination period. Regarding 2-heptanone, no fluctuations were observed, reaching its maximum value in the 31st week, with the O5 area showing the greatest abundance. Furthermore, carobs showed the highest abundance of butanoic acid and hexanoic acid methyl ester in the 31st week. Similarly, propanoic acid, 2-methyl- and 2-methyl butyl esters reached their maximum abundance in the 31st week. However, the highest abundance of propanoic acid, 2-methyl-, ethyl ester was observed earlier (29th week).

**Figure 3:**
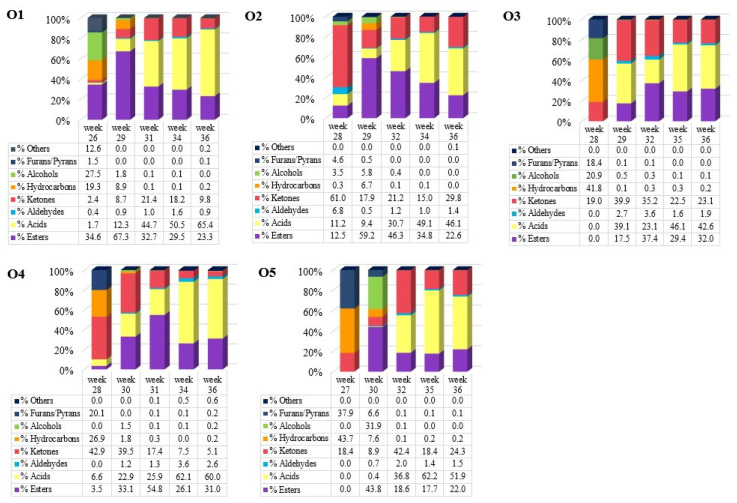
Chemical composition (%) of VOCs per orchard and per examined week.

The different chemical groups play an important role in the determination of carob odor and flavor. Figure 3 shows the distribution of VOCs chemical groups per week of maturation and orchard. The VOCs emitted from the carob pods were classified into chemical classes, with acids (42.6–65.4%) (total area under the curve, AUC) and esters (22.0–32.0%) being the most abundant compounds, followed by ketones (5.1–29.8%). Acids were the main abundant group in the last week of ripening study for all 5 regions, while the percentages of ketones and esters fluctuated in almost all orchards. The other chemical groups, such as aldehydes (<2.6%), alcohols (<0.2%), and furans/pyrans (<0.2%), were present in much lower percentages.

**Figure 4:**
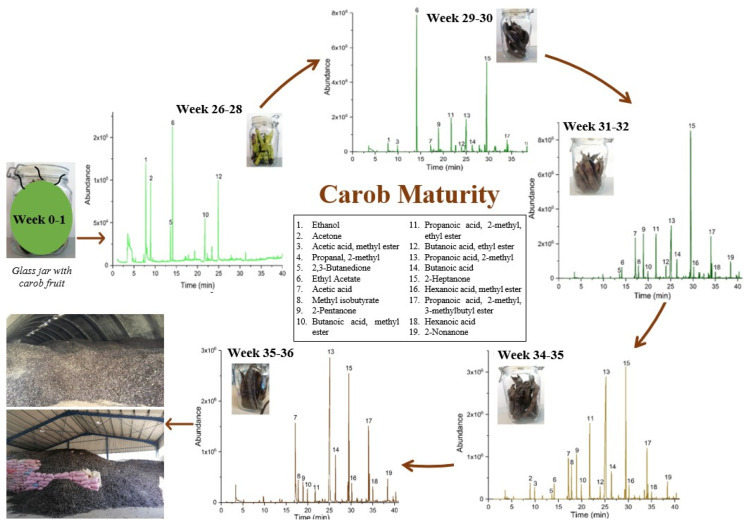
Stages of carob pods maturation through the HS-SPME-GC/MS chromatograms in each examined period (Week 26–36), and the respective influence of the maturation on the emitted volatile compounds.

Figure 4 illustrates the overall HS-SPME-GC/MS procedure of carob ripening from Livadia (O1) with the respective chromatograms of the five study periods of ripening. VOCs fluctuations were observed during ripening, with the maximum emissions emitted at weeks 34–36, as shown in the respective chromatograms. Additionally, the color of carobs during these weeks was dark brown, indicating ripening. Βutanoic acid ethyl ester (12), propanoic acid, 2-methyl- (13), butanoic acid (14), 2-heptanone (15), propanoic acid, 2-methyl-, 3-methyl butyl ester (17), and 2-nonanone (19) were significantly increased in the final stage of the examined period. After being stored in the carob mills, carob fruits were processed to produce several related products (e.g., small- and medium-size kibble nibbles, cubes, seeds, powder, etc.).

It is worth noting that the listed BVOCs in Table 2 along with the corresponding average values of the five regions (O1, O2, O3, O4, and O5) for each examined ripening period were identified in non-roasted carob fruits. Roasting is a thermal procedure that takes place in carob mills to produce carob powder. Some VOCs are released in both the raw carob fruit and carob powder, such as hexanoic acid [33], propanoic acid, 2-methyl-, hexanal, benzaldehyde, o-cymene, limonene [34], as well as furans, and their derivatives (furan, 2-methyl) [35]. Furan levels are raised by roasting and need to be monitored and regulated since they are considered toxic [36]. More specifically, a previous study by Krokou et al. [18] showed that the main chemical group of VOCs in carob fruits are acids, followed by esters, aldehydes/ketones, furans, hydrocarbons, and alcohols. Thus, the present results on non-roasted carob (carob fruit) from the five orchards confirm the earlier findings, as the acids (65.4%) remained the main chemical group (Table 2, Figure 3), followed by esters (32%).

### 2.2. Ripening and Influence on TVOCs, CO_2,_ and Humidity Values

**Figure 5:**
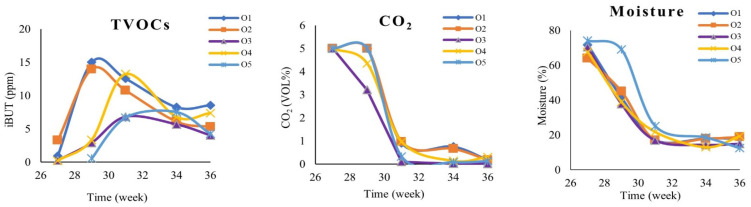
The trends of total VOCs (TVOCs), CO_2_ and humidity per week for the orchards studied (O1, O2, O3, O4, and O5).

Figure 5 shows the trends of TVOCs and CO_2_ emissions per week and for each orchard. Total VOCs show an increasing trend in the first weeks of sampling. After the maximum value was reached, a gradual decline started for all orchards. Additionally, CO_2_ emissions and humidity presented a rapidly declining trend until the 31st week, when they eventually stabilized for all sampling areas. According to Burg et al. [37], CO_2_ as a by-product of the ripening process controls the characteristics of the fruit and can delay ripening.

According to Figure 5, the carobs from regions O1 and O2 presented the highest concentrations of emitted TVOCs in the 29th week, with region O1 having the highest value, followed by O2. In general, for all areas (28th–31st week), a decreasing trend in the TVOCs was noticed. This is attributed to the maturation conditions during the last weeks of the experiment. The potential differences in the observed TVOCs profiles originate from the micro-climate of each region, the altitude, rainfall, and sunshine.

## 3. Materials and Methods

### 3.1. Samples

Carob pods were harvested from five different orchards in the regions of Larnaca and Nicosia (Cyprus), between June and September 2020 (26th to 36th week). More information about the sampling locations is provided in Table 3. After harvesting, the fruits were transferred to the laboratory and carefully cleaned with paper (no water) to remove potential impurities.

### 3.2. HS-SPME-GC/MS Analysis

The HS-SPME technique was applied to extract the VOCs emitted from carob fruits. Carob pods were weighed (315 g), placed in a 1.5 L in-house-made glass jar, and sealed hermetically with a Teflon cap. The sample was left in the jar for 24 h at room temperature prior to the analysis. Then, the 75 μm Carboxen/Polydimethylsiloxane (CAR/PDMS, Supelco) fiber was exposed to the headspace of the jar for 30 min to extract the VOCs. Next, the fiber was thermally desorbed into the GC injector for 1 min at 280 °C in split mode 1:10. Before sampling, the fiber was conditioned according to the instructions of the supplier. Therefore, the injector was set at 300 °C, and the CAR/PDMS fiber was exposed for 30 min. The optimization of the SPME parameters (fiber type, extraction time, desorption time, and temperature) were examined in a previous study [18].

The determination of VOCs was performed using a 7890B GC coupled with a 5977B mass detector (MS) (Agilent, Santa Clara, CA, USA). The VOCs were separated on an SPB-624 capillary column (60 m × 0.25 mm × 1.4 μm film thickness, Sigma-Aldrich Chemie GmbH, Taufkirchen, Germany). Helium was used as the carrier gas at a flow rate of 1.7 mL/min. The initial oven temperature was held at 35 °C for 5 min, then raised to 180 °C with a rate of 4 °C/min, and maintained for 20 min. The MS detector operated under the electron impact (EI) ionization mode at 70 eV with a scan range of 35–350 *m/z*. The temperatures of the quadrupole, ion source, and transfer line were set at 150 °C, 230 °C, and 250 °C, respectively. The identification of compounds was carried out by using the retention times relative to those of analytical standards [18], and a mass spectrum matching the NIST 17 library database.

Each sample was analyzed in triplicate. The respective chromatograms were processed using the AUC (ChemStation software, F.01.03.23.57, Agilent Technologies Inc., Santa Clara, CA, USA). The mean value of AUC was used as displayed in Figure 2 (3 replicates, 5 carob orchards), and Figure 3 (% sum of AUCs per chemical class per sampling week), as well as in Table 2 (% mean of total AUC per VOC from the five examined carob fields).

### 3.3. Determination of Moisture Content

The determination of moisture content in carob fruits was measured following the oven-drying method. The calculation of moisture content was based on the loss of weight; the carobs were weighed and then placed in a forced-air oven (J.P. Selecta, Barcelona, Spain) and heated at 105 °C for 12 h.

### 3.4. Monitoring of Other Gaseous Emissions

A portable photoionization gas detector (PID, Dragger Xam 8000, Dräger Safety AG & Co. KGaA, Lübeck, Germany) was used to monitor the TVOCs and CO_2_ emissions by carobs, after being left for 24 h in a hermetically sealed glass jar (1.5 L).

## 4. Conclusions

A simple, non-invasive, analytical method (HS-SPME-GC/MS) that extracts carob aroma helped to highlight the optimum time of carob ripening. An HS-SPME-GC/MS analysis was applied to reveal carob ripening; variations of VOCs emitted during the ripening stages were noted. This can be attributed to the respective terrain (e.g., temperature, climate, soil composition, topography, cultivar, etc.) of each carob field. Among all the compounds, acids were found to be the major contributors to the perceived carob aroma followed by esters; the abundant presence of isobutyric acid was verified. The methodology is directly applicable to any fruit, even in the field. Thus, unripe and over-ripe carob accumulation in mills can be potentially avoided. The latter contributes to the fruit’s quality and freshness.

## Figures and Tables

**Table 1:** Studies focusing on fruit VOCs ripening.

Sample Type	Analytical Method	Ripening Biomarkers	References
Purple passion fruit (*Passiflora edulis* Sims)	Headspace solid-phase micro extraction gas chromatography/mass spectrometry (HS-SPME-GC/MS)	85 volatile organic compounds (VOCs) (e.g., esters, ketones, alcohols, hydrocarbons, aldehydes, and terpenes) increased at the intermediate and ripe stages (purple color); 49 VOCs were not detected in the immature stage (green color); 11 VOCs (9 esters and 2 alcohols) were found in the ripe stage (symbols for ripe fruits).	[25]
Tamarillo (*Solanum betaceum* Cav.)	HS-SPME-GC/MS	Increase in acids, esters, and terpenoids/decrease in alcohols, phenols; Increase in color index (CI) values and sugars; Color changes from green to red; Softening of the flesh.	[12]
Grape berry	HS-SPME-GC/MS	During the three developmental stages: Increase in aldehydes; Alcohols, ketones, and hydrocarbons compounds did not change; Increase in esters (bourbonene, ethyl geranate, (Z)-butanoic acid, 3-hexenyl ester, and dodecanoic acid ethyl ester are the most dominant esters at ripening stage); Increase in monoterpenes at veraison and ripening stage; Decrease in sesquiterpenes after fruit-set stage.	[26]
Wounding tomato	HS-SPME-GC/MS	40 VOCs were detected and changed at the ripening stage; hexadecane, dodecane, tetradecane,1-chloro, and sulfur compounds (dimethyl disulfide and 2-isobutyl thiazole) were characteristic of the light red developmental stages.	[27]
Strawberry	HS-SPME-GC/MS	Peak intensities of most esters (except for methyl butanoate and methyl hexanoate) and furanones raised during ripening; Red coloration and lower astringent attributes in ripe fruit; Increase in aldehydes and alcohols (e.g., 1-hexanol, 2-hexen-ol, 1-octanol, furaneol, mesifuran, propanal, hexanal, and benzaldehyde), as well as volatile fatty acids; 2-Hexenal was characteristic for the unripe fruit.	[14]
Exotic fruits (avocado, banana, mango, and mangosteen)	Proton-transfer-reaction time-of flight/mass spectrometry (PTR-TOF/MS)	70 VOCs in avocado, 75 VOCs in banana pulp, 85 VOCs in mango, and 53 VOCs in mangosteen were identified; Ripe fruits: methanol, acetaldehyde, and ethanol were the most abundant compounds. Μethanol emissions for avocado, banana, and mangosteen increased, while methanol concentration in mango remained constant. Only for mango fruits, monoterpenes showed a strong discriminating power; Unripe fruits: monoterpenes, sesquiterpenes, cis- and trans-hexenal, and terpenes.	[11]
Mangoes (*Mangifera indica* L. cv.)	PTR-TOF/MS	Potential differentiation of ripe ‘*Tommy Atkins’* and ‘*Keitt’* mangoes; Mono- and sesquiterpenes, methanol, ethanol, acetaldehyde, and esters were related with mango ripeness.	[15]
Peach (*Prunus persica* L. Batsch)	HS-SPME-GC/MS	18 VOCs (lactones, esters, alcohols, aldehydes) were detected. Ripen fruits: increase (↑) of lactones and esters, decrease (↓) of aldehydes. Immature fruits: ↑ (Z)-3-hexen-1-ol and (Z)-3-hexenal.	[28]
Apricot (*Prunus armeniaca* L.)	HS-SPME-GC/MS	47 VOCs (6 aldehydes, 5 alcohols, 7 esters, 5 norisoprenoids, 8 lactones, 10 terpenes, and 6 acids) were reported; ↓ Total aldehydes (hexanal, (Z)-3-hexenal, and (E, Z)-2,6-nonadienal); ↓ Total terpenes (β-myrcene, linalool, α-terpineol, geraniol, and limonene); ↑ Apocarotenoids (β-damascenone, β-ionone, and dihydro-β-ionone); ↑ Total lactones; ↑ Hexyl acetate and (Z)-3-hexenyl acetate.	[29]
Avocado	Gas chromatography/ion mobility spectrometry (GC/IMS)	30 VOCs; Reduction in phenol and acrolein; Increase in chloroform, isoprene, and acetone.	[22]
Mango	e-nose	Ripened fruit: ↑ TVOCs, especially α- and β-pinene, limonene, γ-terpinene, α-terpinolene, β-caryophyllene, α-hmulene, 3-carene, myrcene and α-terpinene; Green fruit: ↑ Hexanal, octanal, and cis-3-hexenol.	[13]
Carob powder	HS-SPME-GC/MS	56 VOCs (acids, esters, aldehydes, ketones, alcohols, furans, alkanes); ↑ Acids (isobutyric acid, most abundant); ↓ Aldehydes and alcohols. Immature stages: Isobutyrate and methyl isobutyrate ester.	[30]

**Table 2:** Mean values $(\frac{Ai}{\sum {Ā}_{i\;(i=3)}})n=5$ × 100 of volatile organic compounds (VOCs) from all fields under study per examined week (mean values = values of area under the curve (AUC) per VOC (3 replicates) derived from the five carob orchards, divided by the total AUC of all VOCs and multiply by 100).

Compound	^a^ W26–28	^b^ SD	W29–30	SD	W31–32	SD	W34–35	SD	W35–36	SD	Reference
**Acids**											
Acetic acid	0.05	0.11	1.34	1.15	5.77	1.91	4.33	1.55	6.06	2.75	* CF [18], ** CP [18,32]
Propanoic acid	0.00	0.00	0.00	0.00	0.06	0.05	0.04	0.03	0.07	0.06	CF [18], CP [18,32,33]
Propanoic acid, 2-methyl-	3.70	4.16	13.19	10.86	22.18	5.05	44.57	7.28	40.54	3.27	CF [18], CP [18,32,33]
Butanoic acid	0.16	0.31	1.03	0.90	2.86	1.15	3.60	0.84	3.97	1.82	CF [18], CP [18,32,33]
Butanoic acid, 3-methyl-	0.00	0.00	0.03	0.03	0.05	0.06	0.05	0.03	0.08	0.09	CF [18], CP [32]
Butanoic acid, 2-methyl-	0.00	0.00	0.25	0.26	0.49	0.24	1.12	0.29	1.20	0.49	CF [18]
Hexanoic acid	0.00	0.00	0.19	0.24	0.85	0.33	1.12	0.34	1.30	0.61	CF [18,33], CP [18,32]
**Alcohols**											
1-Propanol, 2-methyl-	5.31	0.00	0.00	0.00	0.00	0.00	0.00	0.00	0.00	0.00	
Ethanol	9.32	10.09	8.30	11.96	0.20	0.14	0.04	0.04	0.35	0.66	CF [18], CP [18,32]
4-Hexen-1-ol	0.00	0.00	0.00	0.00	0.00	0.00	0.01	0.02	0.11	0.15	
**Aldehydes**											
Acetaldehyde	0.64	1.08	0.33	0.18	0.13	0.04	0.23	0.09	0.46	0.51	CF [18], CP [18,32]
Propanal, 2-methyl-	0.41	0.81	0.27	0.30	0.34	0.26	0.40	0.34	0.88	1.19	CF [18], CP [18,32]
Butanal	0.00	0.00	0.04	0.05	0.00	0.01	0.00	0.00	0.04	0.08	CP [18,32]
Butanal, 3-methyl-	0.24	0.49	0.19	0.25	0.51	0.34	0.49	0.37	1.22	1.66	CF [18], CP [18,32]
Butanal, 2-methyl-	0.16	0.32	0.17	0.25	0.19	0.23	0.11	0.16	0.00	0.00	CF [18], CP [18,32]
Hexanal	0.00	0.00	0.22	0.27	0.33	0.11	0.30	0.18	0.47	0.49	
Heptanal	0.00	0.00	0.00	0.00	0.00	0.00	0.00	0.00	0.04	0.09	
Benzaldehyde	0.00	0.00	0.02	0.02	0.19	0.14	0.13	0.12	0.26	0.22	CF [18], CP [18,32,33]
Octanal	0.00	0.00	0.00	0.00	0.00	0.00	0.01	0.02	0.11	0.21	
Nonanal	0.00	0.00	0.00	0.00	0.11	0.05	0.16	0.05	0.23	0.21	CF [18], CP [18,32]
**Esters**											
Acetic acid, methyl ester	0.00	0.00	0.23	0.32	0.46	0.16	0.81	0.19	1.06	0.61	CF [18], CP [18,32]
Ethyl Acetate	0.85	1.47	6.39	11.10	0.99	0.47	0.59	0.43	0.49	0.55	CF [18], CP [18,32]
Methyl propionate	0.00	0.00	0.00	0.00	0.01	0.01	0.03	0.02	0.06	0.08	CF [18], CP [18,32]
Methyl isobutyrate	0.77	1.00	1.79	1.46	2.75	2.06	3.56	1.26	4.80	2.21	
Propanoic acid, ethyl ester	0.03	0.06	0.06	0.11	0.02	0.02	0.02	0.03	0.01	0.01	
n-Propyl acetate	0.00	0.00	0.06	0.12	0.00	0.01	0.00	0.00	0.00	0.00	
Butanoic acid, methyl ester	0.00	0.00	0.09	0.09	0.81	0.18	1.50	0.28	2.53	2.32	CF [18], CP [18,32]
Propanoic acid, 2-methyl-, ethyl ester	6.32	9.16	14.68	5.29	7.24	4.67	4.32	2.68	3.37	2.86	CF [18], CP [18]
Isobutyl acetate	0.13	0.26	5.46	7.20	0.27	0.24	0.07	0.07	0.14	0.20	CP [18,32]
Butanoic acid, 2-methyl-, methyl ester	0.00	0.00	0.03	0.04	0.06	0.05	0.10	0.05	0.15	0.09	CF [18], CP [18,32]
Butanoic acid, ethyl ester	0.07	0.14	0.79	0.71	1.15	0.48	0.68	0.36	0.98	1.43	CF [18], CP [18,32]
Butanoic acid, 2-methyl-, ethyl ester	0.48	0.69	0.42	0.40	0.04	0.08	0.03	0.05	0.26	0.46	CF [18], CP [18,32]
Butanoic acid, 3-methyl-, ethyl ester	0.09	0.09	0.09	0.03	0.00	0.00	0.00	0.00	0.00	0.00	
Propanoic acid, 2-methyl-, propyl ester	0.00	0.00	0.19	0.24	0.22	0.15	0.10	0.05	0.10	0.06	
1-Butanol, 3-methyl-, acetate	0.74	1.37	0.32	0.39	0.41	0.20	0.33	0.12	0.76	0.56	CF [18],
1-Butanol, 2-methyl-, acetate	0.00	0.00	0.10	0.18	0.10	0.05	0.06	0.02	0.16	0.11	
Propanoic acid, 2-methyl-, 2-methylpropyl ester	0.58	1.16	8.22	7.55	8.57	9.82	2.19	3.15	2.63	4.77	CP [18,32]
Hexanoic acid, methyl ester	0.05	0.11	0.21	0.13	1.18	0.15	1.56	0.14	3.21	3.31	CF [18], CP [18,32]
Butanoic acid, 2-methylpropyl ester	0.00	0.00	0.32	0.36	0.87	0.85	0.26	0.23	0.99	1.60	CP [32]
Propanoic acid, 2-methyl-, 1-methylbutyl ester	0.00	0.00	0.20	0.13	0.67	0.31	0.51	0.43	0.79	0.57	
Butanoic acid, butyl ester	0.00	0.00	0.00	0.00	0.02	0.04	0.00	0.00	0.08	0.16	
Hexanoic acid, ethyl ester	0.06	0.13	0.94	0.88	1.06	0.41	0.49	0.26	0.98	1.57	CP [32]
Butanoic acid, 2-methyl-, 2-methyl propyl ester	0.00	0.00	0.36	0.42	0.44	0.47	0.11	0.13	0.56	0.92	
Isobutyl isovalerate	0.00	0.00	0.03	0.03	0.01	0.02	0.00	0.00	0.04	0.08	
Propanoic acid, 2-methyl-, 2-methyl butyl ester	0.00	0.00	0.96	0.85	5.45	0.81	6.22	1.29	13.19	10.99	CF [18], CP [18,32]
Propanoic acid, 2-methyl-, 3-methyl butyl ester	0.00	0.00	0.62	0.58	1.82	0.74	1.82	0.56	4.29	3.94	
2-Heptanol, acetate	0.00	0.00	0.22	0.00	0.00	0.00	0.00	0.00	0.00	0.00	
Propanoic acid, 2-methyl-, butyl ester	0.00	000	0.43	0.53	0.48	0.43	0.24	0.29	0.27	0.29	
Butanoic acid, 3-methylbutyl ester	0.00	0.00	0.00	0.00	0.17	0.12	0.21	0.08	0.68	0.92	
Butanoic acid, 2-methylbutyl ester	0.00	0.00	0.00	0.00	0.04	0.05	0.01	0.01	0.17	0.23	
Butanoic acid, 2-methyl-, 3-methylbutyl ester	0.00	0.00	0.00	0.00	0.06	0.06	0.07	0.04	0.31	0.40	
Butanoic acid, 2-methyl-, 2-methylbutyl ester	0.00	0.00	0.00	0.00	0.02	0.03	0.00	0.00	0.24	0.34	
Propanoic acid, 2-methyl-, hexyl ester	0.00	0.00	0.15	0.16	0.55	0.43	0.43	0.21	0.86	0.91	
Hexanoic acid, 2-methylpropyl ester	0.00	000	0.21	0.26	0.51	0.59	0.11	0.12	0.56	0.96	
Butanoic acid, 1-methylhexyl ester	0.00	0.00	0.42	0.31	1.08	0.68	0.72	0.32	1.38	0.78	
Isopentyl hexanoate	0.00	0.00	0.00	0.00	0.03	0.04	0.34	0.48	0.16	0.23	
**Ketones**											
Acetone	15.62	13.13	0.54	0.44	0.34	0.19	0.54	0.23	0.60	0.31	CF [18], CP [18,32]
2,3-Butanedione	0.00	0.00	0.37	0.37	0.32	0.15	0.30	0.13	0.42	0.30	CF [18], CP [18]
2-Butanone	0.00	0.00	0.03	0.03	0.05	0.01	0.06	0.02	0.10	0.07	CF [18], CP [18]
2-Pentanone	5.06	9.71	9.02	3.27	6.84	2.51	3.70	1.06	4.06	1.49	CF [18], CP [18]
3-Pentanone	1.31	2.26	0.31	0.31	0.09	0.07	0.01	0.02	0.01	0.01	
2-Hexanone	0.06	0.11	0.14	0.10	0.19	0.08	0.11	0.06	0.25	0.25	CF [18], CP [18]
4-Heptanone	0.58	1.17	0.34	0.30	0.22	0.08	0.09	0.08	0.09	0.11	
2-Heptanone	5.51	11.03	9.97	8.74	17.01	6.78	10.78	4.65	13.74	6.64	CF [18,34]
3-Hepten-2-one	0.00	0.00	0.01	0.02	0.13	0.06	0.11	0.03	0.20	0.18	
2-Octanone	0.25	0.00	1.13	0.00	0.00	0.00	0.00	0.00	0.00	0.00	CF [18]
4-Nonanone	0.15	0.30	0.18	0.11	0.15	0.05	0.04	0.05	0.11	0.13	
2-Nonanone	0.39	0.79	1.18	0.85	2.14	0.61	1.72	0.43	2.84	1.62	CF [18], CP [18]
**Furans**											
Furan, 2-methyl-	0.30	0.59	0.13	0.24	0.03	0.01	0.03	0.02	0.14	0.24	CF [18], CP [18]
Furan, 3-methyl-	20.27	13.66	2.38	3.01	0.00	0.00	0.00	0.00	0.00	0.00	CP [32]
Furan, 2-ethyl-	0.00	0.00	0.00	0.00	0.03	0.03	0.01	0.01	0.10	0.15	
Furan, 2,5-dimethyl-	0.00	0.00	0.00	0.00	0.00	0.00	0.00	0.00	0.07	0.13	
Furan, 2-pentyl-	0.00	0.00	0.03	0.03	0.01	0.01	0.00	0.00	0.13	0.25	
**Hydrocarbons**											
Pentane	10.23	359	0.33	0.47	0.00	0.00	0.00	0.00	0.00	0.00	CF [18], CP [18,32]
Isoprene	20.49	5.92	0.41	0.58	0.00	0.00	0.00	0.00	0.00	0.00	
4-methyl-1,3-pentadiene	0.00	0.00	0.10	0.21	0.00	0.00	0.00	0.00	0.03	0.06	
Octane	0.72	1.27	0.08	0.07	0.16	0.09	0.10	0.12	0.38	0.50	CF [18], CP [18,32]
Styrene	5.86	7.53	4.24	3.24	0.02	0.03	0.00	0.00	0.00	0.00	
Limonene	0.00	0.00	0.02	0.04	0.00	0.00	0.00	0.00	0.15	0.26	CF [18], CP [18,32]
o-Cymene	0.54	0.54	0.12	0.12	0.00	0.00	0.04	0.04	0.00	0.00	CF [18], CP [18,32]
Toluene	5.92	0.00	0.00	0.00	0.00	0.00	0.00	0.00	0.00	0.00	CF [18]
**Others**											
Disulfide, dimethyl	2.51	5.02	0.00	0.00	0.03	0.05	0.12	0.21	0.79	1.43	CF [18], CP [18,32]

^a^ W = Week, ^b^ SD = Standard Deviation, * CF = carob fruit; ** CP = carob powder.

**Table 3:** Geographical locations of carob fruits.

Carob Orchard (Codes)	Village (District)	Coordinates/Altitude	Annual Average Rainfall 2020 (mm) [38]
O1	Livadia (Larnaca)	34°56′49.2″ N, 33°37′50.3″ E (34.947000, 33.630639)	235
O2	Maroni (Larnaca)	34°45′15.5″ N, 33°22′44.3″ E (34.754315, 33.378971)	684
O3	Skarinou (Larnaca)	34°48′36.6″ N, 33°21′21.1″ E (34.810172, 33.355856)	740
O4	Mazotos (Larnaca)	34°48′27.6″ N, 33°29’51.4″ E (34.807672, 33.497615)	235
O5	Kokkinotrimithia (Nicosia)	35°08′32.1″ N, 33°12′15.7″ E (35.142242, 33.204372)	443

The total water inputs of the main dams of Cyprus for the period 2020/2021 was 36.098 million cubic meters (Water Development, Department of Cyprus).

## Data Availability

Not applicable.
